# Identification and molecular characterization of *Corynebacterium xerosis* isolated from a sheep cutaneous abscess: first case report in Mexico

**DOI:** 10.1186/s13104-016-2170-8

**Published:** 2016-07-22

**Authors:** Fernando Hernández-León, Jorge Acosta-Dibarrat, Juan Carlos Vázquez-Chagoyán, Pomposo Fernandez Rosas, Roberto Montes de Oca-Jiménez

**Affiliations:** Centro de Investigación y Estudios Avanzados en Salud Animal, Facultad de medicina veterinaria y Zootecnia, Universidad Autónoma del Estado de México, Km 15.5 Carretera, 50200 Toluca-Atlacomulco, Estado de México Mexico

**Keywords:** *Corynebacterium xerosis*, *Corynebacterium pseudotuberculosis*, Skin abscess, Sheep, *Pld* gene, *RpoB* gene, Case report

## Abstract

**Background:**

*Corynebacterium xerosis* is a commensal organism found in skin and mucous membranes of humans. It is considered an unusual pathogen, and it is rarely found in human and animal clinical samples. Here we describe the isolation of *C. xerosis* from a 4-months-old Pelifolk lamb located in Tesistán, central western Mexico. This microorganism should be considered for differential diagnosis in cutaneous abscessed lesions in sheep, as it represents a zoonotic risk factor for human infection in sheep farms.

**Case presentation:**

The animal exhibited a hard-consistency, 5 cm diameter abscess, without drainage, in the neck. The presumptive clinical diagnosis was caseous lymphadenitis, caused by *Corynebacterium pseudotuberculosis*. Samples were obtained by puncture and cultured in 8 % sheep blood agar under microaerophilic conditions. Colonies were non-haemolytic, brown-yellowish and showed microscopic and biochemical features similar to *C. pseudotuberculosis*, except for the urea test. A multiplex-PCR for the amplification of partial sequences of the *pld*, *rpoB* and intergenic fragment from *16S* to *23S* genes suggested that isolate could be *C. xerosis,* which was later confirmed by sequencing analysis of the *rpoB* gene.

**Conclusions:**

This study shows for the first time isolation and molecular characterization of *C. xerosis* from a clinical sample of an ovine cutaneous abscess in Mexico. This finding highlights the need for differential diagnosis of this pathogen in ovine skin abscesses, as well as epidemiological and control studies of this pathogen in sheep farms.

## Background

*Corynebacterium xerosis* is a commensal organism normally present in skin and mucous membranes of humans and animals [1]. It is considered an unusual pathogen but it is able to cause endocarditis, skin infections and other illnesess [1–6]. Furthermore, it has been shown that human clinical isolates originally identified as *C. xerosis* sometimes correspond to other species of *Corynebacterium* (*C. amycolatum*, *C. freneyi* and *C. hansenii*). This misidentification is due to their close phylogenetic relationship and similar phenotypic characteristics [7–10]. *C. xerosis* grows in colonies of 0.2–1.0 mm in diameter, brown-yellowish in colour, slightly dry in appearance and non-haemolytic when cultured in blood agar. Microscopically, *C. xerosis* is irregularly stained, pleomorphic Gram positive rods presenting club-like ends. Biochemically, they are catalase-positive, and are able to ferment glucose, sucrose and maltose [1, 11–13]. Vela et al. [1] described for the first time the identification of eight isolates of *C. xerosis* from animal clinical samples, using classical microbiological methods as well as molecular genetics. More recently, Palacios et al. [14] showed a more thorough analysis of several *C. xerosis* isolates from animal clinical samples, mostly lesions from swine, including subcutaneous abscesses.

## Case presentation

The present case report is derived from a routine veterinary visit to a 155 animals farm, with two sheep breeds (Pelifolk and Blackbelly), located in Tesistán, municipality of Zapopan, state of Jalisco, Mexico. Facilities from this farm were adapted from previous pig farm, and are still surrounded by pig and cattle farms in the near neighbourhood. The flock was kept on elevated floors to facilitate cleansing and handling, however many roasted sharp objects (such as wire, broken fences, nails, etc.) were present in the pens and cleansing was not being performed properly in the farm at the time of inspection. Feeding was based on locally available feedstuffs, such as; low-quality roughage, swine manure-silage and high-protein commercial feed concentrate. This farm called the veterinary services because of frequent cutaneous abscesses found in the animals, no other complaints related to skin problems were reported. Abscesses do not seemed to affect productivity of the animals, however the presence of such lesions negatively affects animal marketing, and therefore, the owner was interested in finding out about the etiological agent and the recommended treatment to eliminate the disease from the farm. Content of the abscesses was sampled from 31 animals (29 ewes, 1 ram and 1 lamb) by a certified veterinarian and sent to the laboratory for bacteriological diagnosis, in order to find the etiological agents that were causing the problem. Based on appearance of the abscesses, presumptive diagnosis of infection in the farm was *Caseous lymphadenitis* for most animals [4]. However, bacteriological analysis demonstrated that 13 animals were infected with *C. pseudotuberculosis, 1 with Corynebacterium* spp., 2 with *Proteus* spp., 2 with *Streptococcus* spp., and in the remaining 13 cases the pathogen could not be identified. The categorization for the *Corynebacterium* spp. isolate was unclear because the results of the bacteriological and biochemical tests were not concluding.

**Fig. 1:**
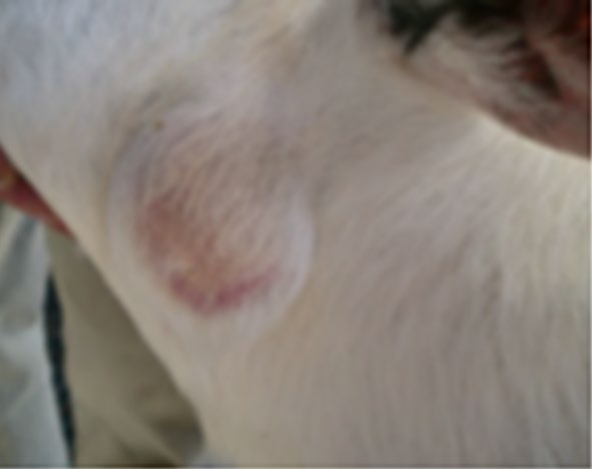
Studied abscess. A hard-consistency abscess without drainage was reported in the neck region of a 4-months-old lamb

**Fig. 2:**
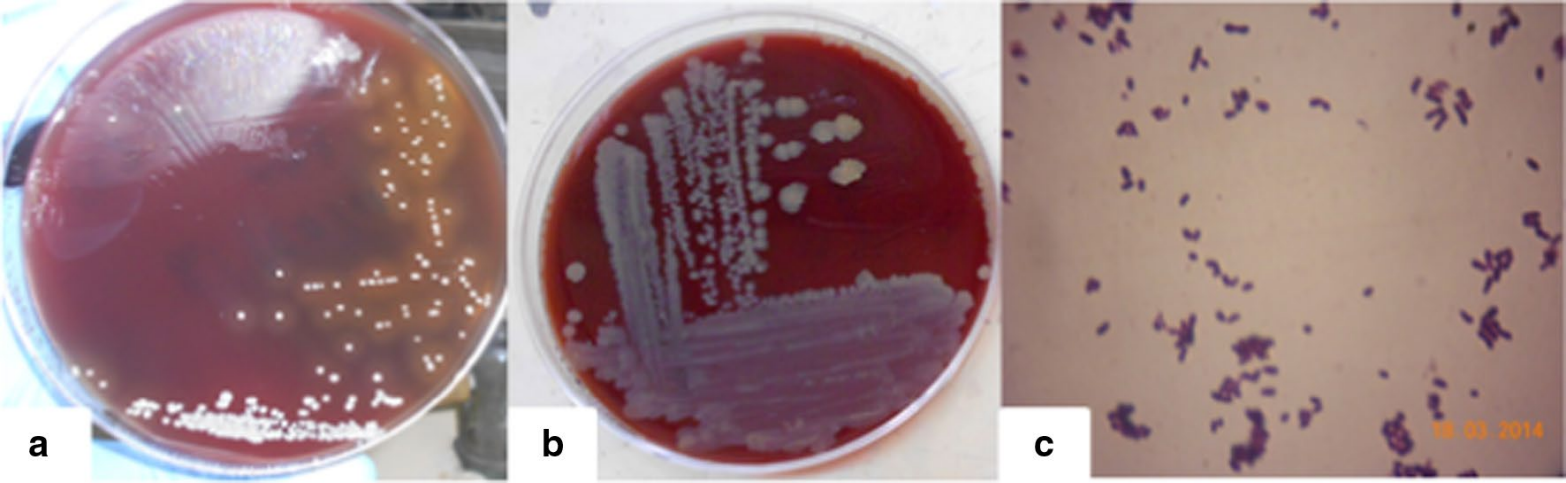
In-vitro Bacteriological culture of C*orynebacterium xerosis and Corynebacterium pseudotuberculosis.* Bacteria were cultured in 8 % sheep blood agar. **a** *Corynebacterium pseudotuberculosis* (ATCC 43924) showed *whitish colonies* with beta haemolysis. **b** *Corynebacterium xerosis* isolate, grew as small *yellowish-brown colonies* without haemolysis. **c** Gram-stained smear preparation of *Corynebacterium xerosis* showing characteristic pleomorphic Gram-positive rods, with club-like ends

**Fig. 3:**
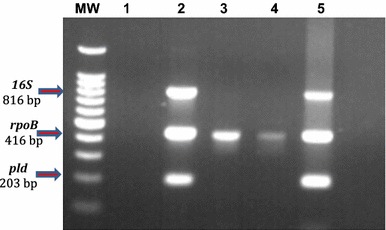
Multiplex PCR. Amplification of partial sequences of *16S rRNA*, *rpoB* and *pld* genes. MW lane: molecular weight marker of 1 Kb Plus DNA Ladder™ (Invitrogen). *Lane 1*: negative control (reaction without template DNA). *Lane 2*: *Corynebacterium pseudotuberculosis* biovar *equi*. *Lanes 3–4*: *Corynebacterium xerosis* isolate (10–0.001 ng of DNA, respectively). *Lane 5*: *Corynebacterium pseudotuberculosis* biovar *ovis*

**Fig. 4:**
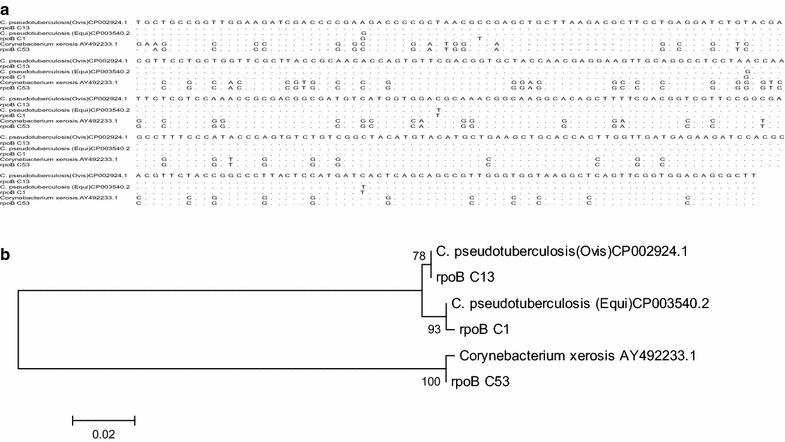
Bioinformatic analysis. **a** Shows the sequence alignment from the sequences used to construct the Phylogenetic tree in (**b**). Three sequences were downloaded from GenBank: *C. pseudotuberculosis* biovar *ovis* (CP002924.1), *C. pseudotuberculosis* biovar *equi* (CP003540.2), and *Corynebacterium xerosis* (AY492233.1). The other three were specimens sent for sequencing (Macrogen, Rockville, MD, USA): rpoB13 (local isolate characterized as C. *pseudotuberculosis,* biovar *ovis*), rpoB C1 (*C. pseudotuberculosis,* biovar *equi*, reference strain ATCC43924) and rpoB C53 (local isolate, characterized as *C. xerosis*). **b** The tree shows the genetic relationship of *Corynebacterium pseudotuberculosis* and *Corynebcaterium xerosis* for the *rpoB* gene. The tree was built from the alignment of partial sequence of the gene. Bootstrap values were obtained generating 1000 random trees, and the strength of each branch is indicated in the respective node

This paper describes the isolation of *C. xerosis* from a 4-months-old Pelifolk (3/4 Pelibuey, 1/4 Suffolk) lamb in good body condition. To the best of our knowledge this is the first report for *C. xerosis* producing a clinical cutaneous abscess in sheep. Informed consent was obtained from the animal’s owner for the publication of the results of the clinical study, including photographic material. The animal exhibited a hard-consistency abscess, without drainage, on the left side of the neck (Fig. 1a). Initial clinical diagnosis suggested caseous lymphadenitis, possibly caused by *C.pseudotuberculosis*. A sample for bacteriological analysis was obtained by puncturing the abscess with a sterile, 5 ml syringe, with a 20 gauge hypodermic needle after cleaning and disinfecting the surface of the abscess. The type of exudate recovered was serous-like and white in appearance. The biological sample was kept at 4 °C until biological characterization at the Centro de Investigación y Estudios Avanzados en Salud Animal (CIESA, km 15.5 Toluca-Atlacomulco road, Toluca, Mexico, z.c. 50200). The time between sampling and processing did not exceed 72 h. Sample was cultured in duplicate, in 8 % sheep blood agar [3] and incubated 24–48 h at 37 °C in aerobic and microaerophilic conditions [1, 14]. Colonies 1.0 mm in diameter, non-haemolytic, yellowish-brown in colour and slightly dry in appearance were observed (Fig. 2a). These morphological characteristics did not correspond to those previously described for *C. pseudotuberculosis,* but they rather seemed colonies from *C. xerosis* [1, 13]. Microscopically (10× magnification; Fig. 2c) bacteria were observed as club-like ended, pleomorphic, irregularly stained Gram-positive rods. Biochemical tests showed the following results: Triple sugar Iron (TSI,−), Lysine Iron agar (LIA,−), Citrate test (CIT,−), Sulphide Indole-Motility (SIM,−), Motility, Indole, Ornitine (MIO,−), Oxidative-fermentative (OF,−) Methyl red (−), Peptone Water (−), Voger Pascaguer (−), Urea (−), Nitrate Broth (+), Trehalose (+), Sucrose (+), Maltose (+) and Glucose fermentation (positive at 37 °C and negative at 42 °C). These results could correspond to a *C. pseudotuberculosis* profile, with the exception of the urea test, which was negative instead of positive, and therefore better coincident with a *C. xerosis* profile [13]. Diagnostic results were not conclusive, therefore, even if the API system is not specific to identify *C. xerosis* [14, 15], we decided to study the isolate with this system, to see if this isolate could be identified as other *Corynebacterium* species. Furthermore, in order to find out if this isolate belongs to other species such as *C. freneyi and C. amycolatum* which can rarely be found in sheep, we tested if the colony was able to grow at 20 °C and if it was able to ferment glucose at 42 °C. The isolate grew fine at 20 °C and did not fermented glucose at 42 °C, both characteristics associated to *C. xerosis* and *C. hansenii* [14], therefore we decided to perform a molecular analysis to find out if we could molecularly identify the isolate as *C. xerosis*. We analysed three loci to increase our diagnostic accuracy. We tested for a gene normally absent in *C. xerosis* but present in *C. pseudotuberculosis*, the *pld* gene [1, 16] where we expected to find no amplicon after PCR test if the isolate was *C. xerosis* in opposition to *C. pseudotuberculosis* which will amplify a 203 bp band; a second locus targeted the amplification of the intergenic spacer region of the 16S–23S rRNA genes (16S–23S) using primers designed for *C. pseudotuberculosis*, which have been reported not to work for *C. xerosis* [16], and we would also expect no amplification band for *C. xerosis* and a 816 bp band for *C. pseudotuberculosis*. Finally we performed PCR amplification and sequencing of the *rpoB* gene (446 bp), which has previously been reported to differentiate species of *Corynebacterium* [14, 16]. DNA extraction was carried out using a commercial kit (KAPA Express Extract) following the manufacturer’s protocol. A Multiplex-PCR technique for amplification of partial sequences of the *pld*, 16S–23S and *rpoB* genes was performed using the protocol published by Pacheco in 2007 [16]. Reactions were carried out using a commercial multiplex PCR kit (QIAGEN Multiplex PCR) following the manufacturer’s specifications. The PCR analysis included the following samples: The isolate to be characterized, the putatively *C. xerosis* isolate, and two *C. pseudotuberculosis* (one local isolate previously characterized as biovar *ovis* and, a reference strain, ATCC 43924, biovar *equi*) used as controls. As expected for the putative *C. xerosis*, a single PCR 446 bp amplicon band, corresponding to the *rpoB* gene fragment was amplified, and the intergenic 16S–23S gene and *pld* gene fragments were not amplified. Also as expected, both *C. pseudotuberculosis* strains showed three bands of 203, 446 and 816 bp, corresponding to genes *pld*, *rpoB* and *16S*, respectively (Fig. 3). *RpoB* gene amplicons of all the three samples were purified using the Promega purification kit (Wizard^®^ SV Gel and PCR Clean-Up System) and were sent for automatic sequencing by Macrogen (Rockville, MD, USA). Multiple sequence alignments obtained from a BLAST (NCBI) were analysed along with our isolate sequences using Clustal W analysis from Mega 6.0.6 (Fig. 4). Phylogenetic analysis was performed using the neighbour-joining method (MEGA software 6.0.6). Bootstrap values were obtained generating 1000 random trees. Phylogenetic analysis also included the sequences from the *rpoB* gene from *C. xerosis* (GenBank AY492233.1), *C. pseudotuberculosis* biovar *ovis* (GenBank CP002924.1) and *C. pseudotuberculosis* biovar *equi* (GenBank CP003540.2). It was possible to observe different phylogenetic groups that corresponded to particular species of *Corynebacterium*. These results, confirm that this study’s isolate (*rpo*B C53) is *C. xerosis* (Fig. 4).

Genotyping techniques such as PCR as well as sequence analysis of the *rpo*B C53 isolate in this study contributed greatly towards a correct species identification, which was originally misleading when using phenotypic microscopic and biochemical features. *Corynebacterium xerosis* has been previously reported in clinical samples of human cases in lesions in endocarditis, pneumonitis, osteomyelitis and skin infections, especially in immunocompromised patients [3, 12]. It has also been found in animal clinical samples, for instance, in goat liver lesions (suspected pseudotuberculosis), and cow milk, from animals presenting mastitis. In swine, *C. xerosis* has been isolated from lesions in different tissues such as liver, kidney, lung, spleen, joints, as well as from subcutaneous abscess. Additionally, *C. xerosis* has been isolated from ovine clinical samples of uterus, in a case of abortion, and from lung tissue, from animals presenting respiratory problems [1, 14]. These isolates were identified and characterized by amplification of the *rRNA 16S*–*23S* gene using PCR–RFLP. However, in those studies, it was not possible to obtain a band pattern clear enough to differentiate *C. xerosis* from other species of *Corynebacterium*. The authors also conducted an analysis and sequence comparison of genes *16S* and *rpoB*, which allowed them to differentiate *C. xerosis* from other species, genetically similar to *Corynebacterium* [1, 14].

As mentioned above, this study was conducted based on the presence of the *rpoB* gene, which has been reported by other authors as the gene of choice for phylogenetic analysis of the genus Corynebacterium, as it presents high polymorphism, even greater than the intergenic spacer region of the 16S–23S rRNA genes [17, 18]. It is known that cutaneous abscesses in sheep (caseous lymphadenitis) is caused by *C. pseudotuberculosis* and its main factor of virulence and pathogenicity is the exotoxin phospholipase D, encoded by the *pld* gene and expressed in the cell membrane of the bacterium [19]. This exotoxin is a permeability factor that promotes hydrolysis of the ester bonds in sphingomyelin in mammalian cell membranes, possibly contributing to the spread of bacteria from the initial site of infection to secondary sites through the lymphatic system to regional ganglia, and it seems to be involved in the reduction of macrophage viability following infection [19]. The exotoxin also causes dermonecrotic lesions [20, 21]. However, this exotoxin has not been reported in *C. xerosis* as a pathogenic toxin that could contribute to the development of abscesses. This highlights the importance of carrying out further research regarding the mechanisms of infection present in this *Corynebacterium* species.

A major point of discussion of epidemiological nature, include the fact that the ovine production system, where the sample was obtained from, was previously used for swine, a species where *C. xerosis* has been reported as a common pathogen [14]. Moreover, one of the main ingredients of sheep diet includes pig by-products (*swine manure*-*silage*), which could eventually have active pathogens, maybe including *Corynebacterium* spp. In addition, sharp objects such as metallic rods, nails and wires, are widespread all over the facilities, which increase the chance of injury of the animals, opening a way of entrance of *Corynebacterium* spp. to the organism including *C. xerosis*. All these factors may have contributed to the presence of *C. xerosis* inside the facility. No further studies were conducted, however recommendations for the owner was to debride and disinfect abscesses from all animals, in a infection containing area (to avoid spread of the pathogens), reinforce pens cleansing and remove all sharp edges and objects from the fences, feeders and floors. Since *C. xerosis,* as well as *C. pseudotuberculosis,* are potentially zoonotic microorganisms, recommendations were made to the farmer to extreme precautions during handling of animals and waste produced by the farm to prevent human and animal infection and the possible widespread of the microorganism to other farms.

## Conclusions

Here we report the isolation and molecular characterization of *C. xerosis* from a clinical sample from a sheep cutaneous abscess for the first time in Mexico. These results encourage further exploration of the factors responsible for *C. xerosis* causing cutaneous abscesses.

## Abbreviations

*C. pseudotuberculosis*: *Corynebacterium pseudotuberculosis*
*C. xerosis*: *Corynebacterium xerosis*
*C. amycolatum*: *Corynebacterium amycolatum*
*C. freneyi*: *Corynebacterium freneyi*
*C. hansenii*: *Corynebacterium hansenii*
*C. ulcerans*: *Corynebacterium ulcerans*
CIESA: Centro de Investigación y Estudios Avanzados en Salud Animal
TSI: triple sugar iron
LIA: lysine iron agar
CIT: citrate test
SIM: sulfide indole-motility
MIO: motility, indole, ornitine
OF: oxidative-fermentative
